# A Korean Patient With Leber Congenital Amaurosis and a Homozygous *RPE65* Variant Originating From a Paternal Uniparental Isodisomy

**DOI:** 10.1002/mgg3.70060

**Published:** 2025-01-21

**Authors:** Hane Lee, Dongseok Moon, Rin Khang, Go Hun Seo, Chang Ki Yoon, Un Chul Park, Kyu Hyung Park, Eun Kyoung Lee

**Affiliations:** ^1^ Division of Medical Genetics 3billion Inc Seoul Korea; ^2^ Department of Ophthalmology Seoul National University College of Medicine, Seoul National University Hospital Seoul Korea

**Keywords:** exome sequencing, gene therapy, Leber congenital amaurosis, *RPE65*, uniparental Isodisomy

## Abstract

**Background:**

Leber congenital amaurosis (LCA), the most severe form of inherited retinal dystrophy, is a rare, heterogeneous, genetic eye disease associated with severe congenital visual impairment. *RPE65,* one of the causative genes for LCA, encodes retinoid isomerohydrolase, an enzyme that plays a critical role in regenerating visual pigment in photoreceptor cells.

**Methods:**

Exome sequencing (ES) was performed on a patient with suspected LCA.

**Results:**

Here, we report a 33‐year‐old male patient diagnosed with *RPE65*‐related LCA caused by uniparental isodisomy (UPiD) who received gene therapy as treatment, fourth patient to receive it in Korea. His fundus examinations showed salt‐and‐pepper retinal dystrophy, with diffuse extinguished signal on fundus autofluorescence and attenuated amplitude on electroretinogram. A homozygous frameshift variant NM_000329.3:c.1067del (p.Asn356MetfsTer17) in *RPE65* was identified by ES with the entire chromosome 1 proving to be paternal UPiD. Within 5 months after the molecular diagnosis, the patient was treated with subretinal voretigene neparvovec (VN) therapy and is being followed up for prognosis.

**Conclusions:**

To our knowledge, this patient is the first UPiD case to receive VN treatment. Performing ES as a first‐tier test was favourable because it allowed to identify UPiD that needed to be detected in addition to the disease‐causing variant.

## Graphical abstract

A 33‐year‐old male diagnosed with *RPE65*‐related Leber congenital amaurosis became the fourth patient in South Korea to receive subretinal voretigene neparvovec gene therapy after being found by exome sequencing (ES) to have a homozygous pathogenic variant due to paternal uniparental isodisomy (UPiD). This patient is the first documented case of UPiD receiving VN treatment, highlighting the value of ES as a first‐tier test for identifying both UPiD and causal variants.

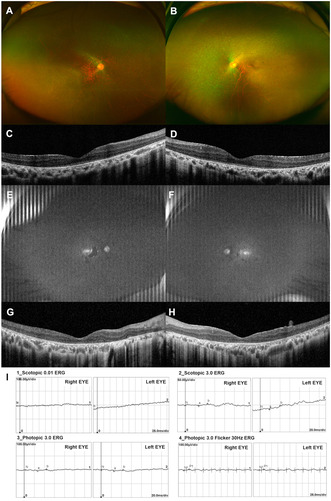

## 1 Introduction

Leber congenital amaurosis (LCA) is the earliest onset and most severe form of inherited retinal dystrophies (IRD) (den Hollander et al. 2008). It is characterized by severe vision loss from birth, nystagmus, sluggish pupillary responses, and an extinguished electroretinogram (den Hollander et al. 2008). The prevalence of LCA is estimated from 1.20 to 2.37 per 100,000 (Han et al. 2023). To date, 25 genes when mutated have been identified to be responsible for LCA (Retinal Information Network, http://sph.uth.edu/retnet/sum‐dis.htm). LCA is a Mendelian disease, most of which are inherited in an autosomal recessive manner, although some autosomal dominant families have been reported.

The *RPE65* gene is one of the causative genes for LCA that has received the most scrutiny due to the approval of first‐in‐class gene therapy using a recombinant adeno‐associated virus (AAV) (voretigene neparvovec [VN]; Luxturna, Spark Therapeutics) (Russell et al. 2017). It encodes retinoid isomerohydrolase, which converts all‐*trans*‐retinyl ester to 11‐*cis*‐retinol, enabling a new photoisomerization event. Mutations in *RPE65* reduce the level of 11‐*cis*‐retinol and cause retinyl esters to accumulate in the retinal pigment epithelium (RPE), blocking the visual cycle (Han et al. 2023).

Uniparental disomy (UPD) is a rare condition in which an individual with a diploid genome carries either two homologs of a pair of chromosomes from one parent (uniparental heterodisomy) or two copies of a single chromosome from one parent (uniparental isodisomy [UPiD]). Identifying UPD is important because in recessive monogenic diseases, even if one parent is a carrier, UPD can form a homozygous allele and lead to recessive monogenic disease. There have been few reports of IRD caused by UPD on chromosomes 1, 2, 4, 6, 8, and 14 (Motta et al. 2021; Pentao et al. 1992; Riveiro‐Alvarez et al. 2007; Rivolta, Berson, and Dryja 2002; Souzeau et al. 2018; Tachibana et al. 2022; Thompson et al. 2002). However, there are only a few cases of *RPE65*‐related LCA caused by UPD to date (Motta et al. 2021; Stepanova et al. 2023; Thompson et al. 2002).

Herein, we report a case of paternal UPiD on chromosome 1 including the *RPE65* gene, resulting in a *RPE65*‐related LCA. The father is an *RPE65* heterozygous carrier, and the mother does not carry any pathogenic variant. Detailed clinical description of the patient as well as the genetic analysis that identified the disease‐causing variant and UPiD are presented.

## 2 Materials and Methods

### 2.1 Ethical Compliance

The study was approved by the Institutional Review Board of Seoul National University Hospital (2405‐032‐1534) and adhered to the tenets of the Declaration of Helsinki.

### 2.2 Subjects

The medical history and comprehensive ophthalmological exams of a 33‐year‐old male proband with LCA were collected from the Inherited Retinal Disease Clinic at Seoul National University Hospital. To identify the genetic cause, buccal swab specimens of the proband and his parents were collected. Written informed consent for the use of personal medical and genetic data for scientific purposes was obtained.

### 2.3 Genetic Testing

A diagnostic IRD panel by exome sequencing (ES) was performed on the proband. Genomic DNA was extracted using AccuBuccal DNA Preparation Kit (AccuGene). Exome capture was performed with xGen Exome Research Panel v2, supplemented with xGen human mtDNA panel and xGen Custom Hyb Panel v1 (Integrated DNA Technologies, Coralville, Iowa, USA), and sequencing was performed using NovaSeq 6000 (Illumina, San Diego, CA, USA) as 150 bp paired‐end reads. Sequencing data were analyzed as previously described (Seo et al. 2020). Briefly, the sequence reads were aligned to the GRCh37 human reference genome and the revised Cambridge Reference Sequence (rCRS) of the mitochondrial genome using BWA‐MEM2 (v2.2.1) to generate BAM files (Li 2013). Variants were called following the GATK best practices (GATK v4.2.14) for single nucleotide variants (SNV) and small insertions/deletions (INDEL) (McKenna et al. 2010). 3bCNV, an internally developed tool (manuscript in preparation), was used to call copy number variants (CNV). The variant analysis was carried out using an internally designed system, EVIDENCE v4 (Seo et al. 2020), that incorporates Ensembl Variant Effect Predictor for annotation and the American College of Medical Genetics and Genomics (ACMG) guideline for variant classification (Richards et al. 2015). Variants were filtered out if too common in the population (minor allele frequency > 5% in gnomAD or > 1% internally) or synonymous with spliceAI score < 0.2. Rare non‐synonymous variants were filtered by the 402 IRD genes, and the final variant list was manually reviewed by medical geneticists and physicians.

### 2.4 Sanger Confirmation and Analysis of Uniparental Isodisomy

Sanger sequencing was performed on the proband's genomic DNA. PCR amplification and Sanger sequencing were performed following the standard protocol using PCR Master Mix Kit (ThermoFisher Scientific, Waltham, MA, USA) and SeqStudio Genetic Analyzer (Applied Biosystems, Foster City, CA, USA). The sequencing results were analyzed with Sequence Scanner version 1.0 (Applied Biosystems, Foster City, CA, USA).

AutoMap v1.2 was used to detect regions of homozygosity (ROH) from VCF files (Quinodoz et al. 2021). Depth‐of‐coverage was examined within the ROH to determine whether it was a copy‐number‐neutral ROH. To determine the parental origin of the potential UPiD, parental samples were subject to ES. Joint genotyping was performed by GATK v4.2.14, and the proband's genotype was compared to the parental genotype within the ROH to detect variants that were violating the Mendelian's law of inheritance. Variant positions for which both parents had at least one alternate allele were considered uninformative and removed from the analysis.

## 3 Results

### 3.1 Case Report

The patient was a Korean male who was referred to the clinic at age 18 years with low vision and night blindness. He was born to healthy non‐consanguineous parents with uncomplicated delivery, and there were no other affected family members with IRD. He had no other health problems. Parents reported that the patient could not see well in the dark since the age of one year.

On initial examination, the best‐corrected visual acuity was 20/50 in both eyes. Refractive error was −5.0 Dsph = −2.75 Dcyl × A180 in the right eye (OD) and − 4.5 Dsph = −3.0 Dcyl × A180 in the left eye (OS). A slit lamp examination revealed no specific findings and nystagmus was not present. Fundus examination revealed narrow retinal vessels and diffuse granular retinal dystrophy with lack of bone spicule pigmentation. The electroretinogram was extinguished, and the remaining visual field was central 10°–15° with the II4e target on Goldmann perimetry. Based on the aforementioned clinical findings, the clinical diagnosis of LCA was made.

**FIGURE 1:**
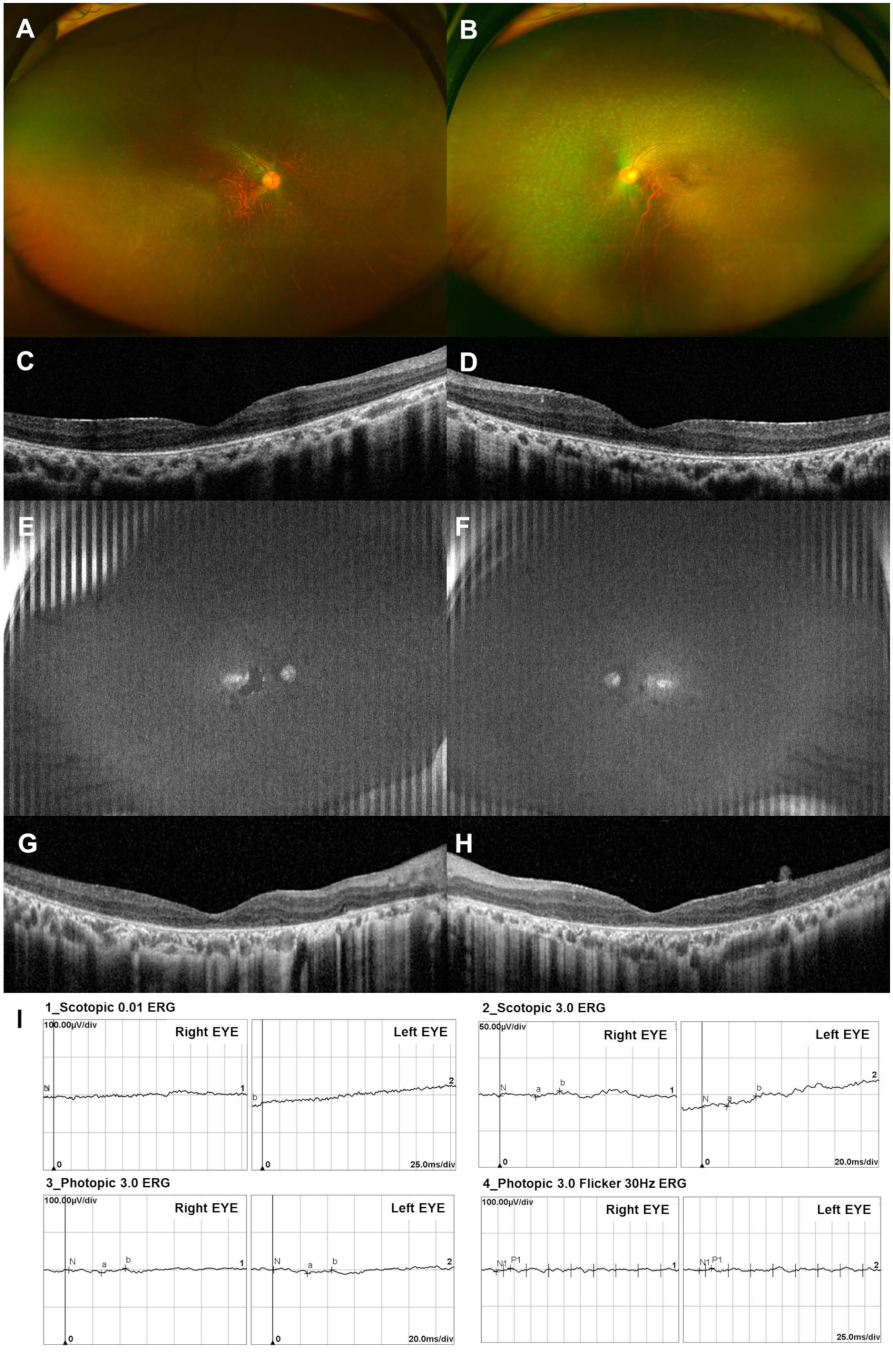
(A, B) Ultra‐widefield fundus photographs show salt‐and‐pepper retinal dystrophy and no apparent bone spicule pigmentation. (C, D) Optical coherence tomography reveals generalized loss of the ellipsoid zone outside of the foveal area. (E, F) Ultra‐widefield fundus autofluorescence reveals nearly absent autofluorescence signal with abnormal foveal hyperautofluorescence and patchy hypoautofluorescent lesions. (G, H) Optical coherence tomography reveals diffuse loss of the ellipsoid zone with a small residual ellipsoid zone at the fovea center, disorganization of layer stratification, and thin epiretinal membrane. (I) Electroretinogram shows extinguished rod and cone responses.

At the age of 23, his visual acuity was 20/50 OD and 20/63 OS. Ultra‐widefield fundus photographs revealed salt‐and‐pepper retinal dystrophy with substantial RPE mottling and fine pigment clumping (Figure 1A,B). Optical coherence tomography (OCT) showed generalized disruption of foveal microstructures, with a relatively preserved central ellipsoid zone in both eyes (Figure 1C,D). The remaining visual field was central 5°–10° with the II4e target.

At the age of 33, his visual acuity was 20/100 in both eyes. The fundus findings were comparable, but the RPE atrophy and pigment clumping were more progressed. Fundus autofluorescence demonstrated diffuse extinguished autofluorescence signal with abnormal foveal hyperautofluorescence and patchy hypoautofluorescent lesions in both eyes (Figure 1E,F). OCT revealed extensive disruption of foveal microstructures, with a small area of preserved ellipsoid zone subfoveally in both eyes (Figure 1G,H). The electroretinogram revealed extinguished rod and cone responses in both eyes (Figure 1I). The remaining visual field was parafoveal island OD and central 5° OS with the III4e target. He was approved for the subretinal VN gene therapy within 5 months of the molecular diagnosis.

### 3.2 Diagnostic Finding From IRD Panel by ES

**FIGURE 2:**
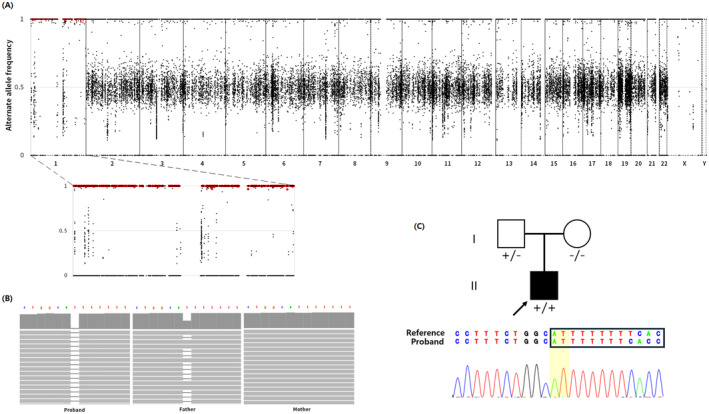
(A) Alternate allele frequency distribution across all chromosomes in the proband (top) and a zoomed‐in view of chromosome 1 (below). *X*‐axis is the chromosome location and *Y*‐axis is the alternate allele frequency. Each dot represents a single nucleotide variant (SNV). Homozygous SNV that were inherited only from the father are represented by red dots. (B) Integrated Genome Viewer (IGV) screenshots of the variant and flanking region are shown for all three individuals. (C) Pedigree shows that the proband denoted with an arrow is homozygous for the variant, father is heterozygous, and mother is wild type. Sanger sequencing trace of the homozygous variant is shown for the proband.

Within the IRD panel, eight rare non‐synonymous SNV/INDEL were identified. There were no rare CNV spanning an IRD gene. There was only one pathogenic variant: a homozygous frameshift variant, 1‐68438247‐AT‐A (GRCh37); NM_000329.3:c.1067del; (p.Asn356MetfsTer17) in the *RPE65* gene (OMIM: 180069). Loss‐of‐function (LOF) is a disease mechanism for the autosomal recessive forms of the *RPE65*‐related disorders (den Hollander et al. 2008). There is a ClinVar (Accession ID: VCV000098821) entry for the identified variant with multiple submissions. It has also been reported in the literature multiple times (Hanein et al. 2004; Marlhens et al. 1997). The variant is a rare variant observed only as heterozygous in the gnomAD v4.0.0 database 5 times. There is at least one report of two siblings with autosomal recessive LCA who carry the variant *in trans* with another pathogenic variant (Marlhens et al. 1997). The variant is predicted to result in a premature termination codon, nonsense‐mediated mRNA decay, and a loss or disruption of normal protein function. There are many pathogenic variants predicted as LOF reported downstream of the variant. The variant was confirmed by Sanger sequencing (Figure 2B,C).

### 3.3 Confirmation of Paternal Uniparental Isodisomy

Nearly all of chromosome 1 was identified as ROH by AutoMap v1.2 (Figure 2A). There were no copy‐number‐loss within this region, suggesting that the ROH was because of UPiD. The proband's genotype of 4608 high‐quality (GATK QUAL score > 500 and PASS‐ed) SNV on chromosome 1 was compared to the parental genotype. Of these, 3593 variant positions were uninformative and removed as both parents carried the alternate allele. Of the remaining 1015 variant positions, 976 positions were homozygous in the proband, and except for one position, 975 positions were heterozygous or homozygous alternate in the father whereas homozygous reference in the mother, meaning that the alternate alleles were only being inherited from the father across the entire chromosome, and therefore, the ROH is because of paternal UPiD. The variant positions that were heterozygous in the proband were manually inspected and determined to be technical false calls.

## 4 Discussion

Here, we report a 33‐year‐old Korean male patient with *RPE65*‐related LCA. IRD panel genetic testing revealed a homozygous pathogenic frameshift variant in the *RPE65* gene that is associated with autosomal recessive LCA. Because ES was utilized for the IRD panel testing, we were able to call ROH across the genome and detect the single ROH involving the entire chromosome 1. ROH can arise from heterozygous deletions, consanguinity, or UPiD. In our patient, since the ROH involved the entire chromosome and was limited to a single chromosome, consanguinity was unlikely. Additionally, the absence of depth‐of‐coverage changes ruled out deletions, making UPiD the most plausible explanation. Subsequently, ES of both parents revealed that the homozygosity corresponded with the father's genotype across the entire chromosome 1, suggesting that the UPiD was of paternal origin. If ES were not performed on the backend, it would not have been possible to suspect UPiD until genotyping of both parents, which would have revealed only the father carrying the same variant as heterozygous and the mother being a wild type.

A case of *RPE65*‐associated retinopathy due to UPiD on chromosome 1 was first reported by Thompson et al. (2002). The patient had a complete paternal UPiD leading to homozygosity for a splice site mutation (IVS1+5G>A) in the *RPE65* gene. Later, Motta et al. (2021) described a case with *RPE65*‐associated LCA caused by a segmental maternal UPiD leading to a homozygous pathogenic variant in *RPE65* (c.1022T>C:p.Leu341Ser). Furthermore, Stepanova et al. (2023) recently analyzed *RPE65* variants in Russian patients and reported two cases of UPiD with paternal and maternal origin, respectively, resulting in a homozygous variant (c.304G>T:p.Glu102*) in *RPE65* gene. Including our patient, none of the patients reported with UPiD‐driven homozygous *RPE65* pathogenic variants had phenotypic differences compared to those with simple homozygous or compound heterozygous variants (Thompson et al. 2002). This confirms that there are no paternally or maternally imprinted genes on chromosome 1 which result in a major phenotype change or a phenotype expansion.

Several cases of UPiD‐associated IRD have been described, including Stargardt disease with *ABCA4* gene (Riveiro‐Alvarez et al. 2007), cone dystrophy with *TULP1* gene (Roosing et al. 2013), retinitis pigmentosa with *USA2A* (Rivolta, Berson, and Dryja 2002) or *MERTK* (Thompson et al. 2002) genes, and LCA with *CRB1* gene (Stone 2007). Knowing the presence of UPiD is important for genetic counseling of families with recessively inherited diseases not only because an unaffected heterozygous carrier and a partner with a wild‐type allele at the disease locus can give birth to an affected homozygous offspring but also because the recurrence risk changes dramatically from 25% to nearly zero when the genetic cause of autosomal recessive disease is because of UPiD. ES is a powerful method to identify both the disease‐causing variant and UPiD simultaneously.

VN is a recombinant AAV 2 vector containing human *RPE65* complementary DNA that enables RPE cells to produce the retinoid isomerohydrolase, thus allowing the restoration of the visual cycle. Following confirmation of efficacy and safety through an open‐labeled, randomized, controlled phase 3 trial, VN was approved for gene augmentation therapy in *RPE65*‐associated retinal dystrophy in 2017 (Russell et al. 2017). All *RPE65*‐associated retinal dystrophy patients who have sufficient viable retinal cells with confirmed biallelic mutations in the *RPE65* gene, including patients with UPiD, are eligible for VN therapy. However, to the authors' knowledge, there have been no reported case of VN treatment in *RPE65*‐associated LCA because of UPiD. The patient described in the present study underwent bilateral subretinal injections of VN. We anticipate reporting short‐ and long‐term outcomes once he has sufficient postoperative follow‐up.

## Author Contributions

**Hane Lee:** conceptualization, formal analysis, methodology, writing – original draft, writing – review and editing. **Dongseok Moon:** writing – original draft and formal analysis. **Rin Khang and Go Hun Seo:** formal analysis, writing – review and editing. **Chang Ki Yoon, Un Chul Park, and Kyu Hyung Park:** writing – review and editing. **Eun Kyoung Lee:** conceptualization; writing – original draft, writing – review and editing; project administration; funding acquisition.

## Conflicts of Interest

The authors declare no conflicts of interest.

## Data Availability

The data that support the findings of this study are openly available in ClinVar at https://www.ncbi.nlm.nih.gov/clinvar/, reference number VCV000098821.14.
